# Impact of the Free-Pelvis Innovation in Very Rigid Braces for Adolescents with Idiopathic Scoliosis: Short-Term Results of a Matched Case-Control Study

**DOI:** 10.3390/children9060871

**Published:** 2022-06-11

**Authors:** Stefano Negrini, Fabrizio Tessadri, Francesco Negrini, Marta Tavernaro, Andrea Zonta, Fabio Zaina, Sabrina Donzelli

**Affiliations:** 1 Department of Biomedical, Surgical and Dental Sciences, University “La Statale”, 20122 Milan, Italy; stefano.negrini@unimi.it; 2 IRCCS Istituto Ortopedico Galeazzi, 20161 Milan, Italy; 3 Orthotecnica, 38014 Trento, Italy; fabriziotessadri@gmail.com; 4 Department of Biotechnology and Life Sciences, University of Insubria, 21100 Varese, Italy; 5 Istituti Clinici Scientifici Maugeri IRCCS, 21049 Varese, Italy; 6 ISICO (Italian Scientific Spine Institute), 20141 Milan, Italy; marta.tavernaro@isico.it (M.T.); andrea.zonta@isico.it (A.Z.); fabio.zaina@isico.it (F.Z.); sabrina.donzelli@isico.it (S.D.)

**Keywords:** Adolescent idiopathic scoliosis, brace, rehabilitation

## Abstract

We introduced pelvis semi-rigid material (ethylene vinyl acetate) (Free-Pelvis) to improve the comfort and adaptability of very rigid braces (VRBs) for adolescents with idiopathic scoliosis (AIS), but this can also negatively impact the corrective forces on the trunk. Study Design: This was a matched retrospective cohort study. The inclusion criteria were AIS, age 10–16, VRB 23 h/day, X-rays available, primary curve 36°–65°, and angle of trunk rotation 7–23°. The cases were Sforzesco VRB with Free-Pelvis (FPB). The controls included classical Sforzesco VRB matched for Risser (range 0/4), menarche age (10/15), weight (33.5/83 kg), height (140/180 cm), BMI (13.5/29 kg/sqm), aesthetics (TRACE 4/12), plumbline distances (S1: −60/35; C7 + L3: −10/115 mm), and referred brace use (22/24 h/day). Statistics: predictors of the results have been tested with linear and logistic regression according to the outcome variable type. We performed logistic regression for improved vs. worsened. The explanatory variable was brace type. We included 777 VRB and 25 FPB, age 13 ± 1, 47° ± 8° Cobb, and 11% men. The few baseline statistical differences were not clinically relevant. We achieved in-brace corrections of 15.2° ± 7.7° and 17.4° ± 6.5° for VRB and FPB, respectively (*p* = 0.21); out-of-brace corrections at 5 ± 2 months were 7.8° ± 0.2° for VRB and 8.1° ± 1.3° for FPB (*p* = 0.83). The type of brace did not influence the Cobb angle at either time interval or affect the odds of improvement. Free-Pelvis innovation, introduced to improve comfort and adaptability, does not change the in-brace or short-term results of classical VRB and consequently can be safely applied.

## 1. Introduction

Idiopathic scoliosis is the most common spinal deformity during growth [1]. Good quality studies showed the efficacy of bracing to treat adolescents with idiopathic scoliosis (AIS) [2,3], and a Cochrane Review summarized the current evidence [4]. Recently, the International Society of Scoliosis Orthopaedic and Rehabilitation Treatment (SOSORT) developed the first classification of braces for idiopathic scoliosis, together with the Scoliosis Research Society (SRS), International Society of Prosthetics and Orthotics (ISPO) and Pediatric Orthopedic Society of North America (POSNA), and with the support of the European Society of Physical and Rehabilitation Medicine (ESPRM) [5]. The classification introduces rigidity as a critical characteristic to distinguish braces, with three categories: elastic, rigid, and very rigid [5]. It has been proposed that the changes in the rigidity of braces can explain the efficacy in different populations, with biomechanically stronger orthoses allowing the treatment of worse AIS [6].

**Figure 1:**
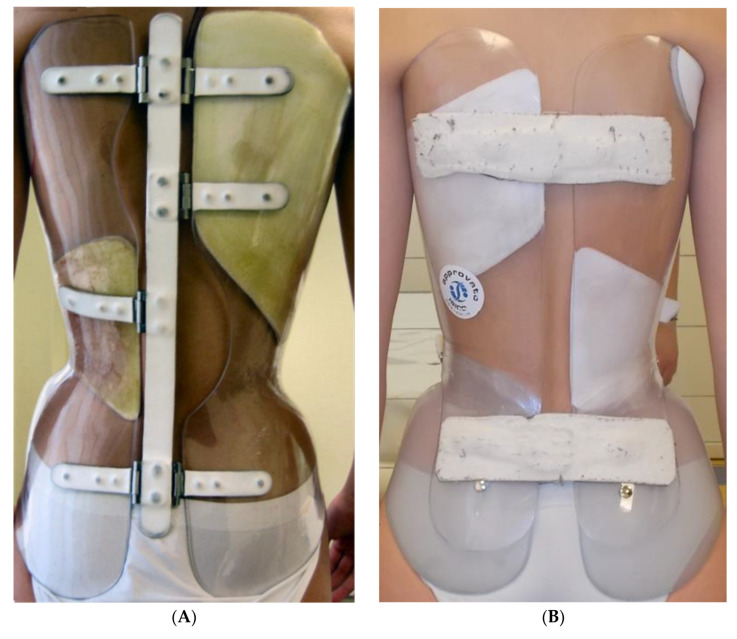

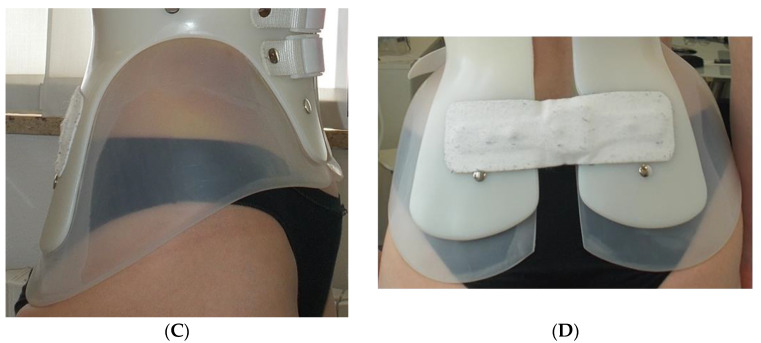
The two types of very rigid braces compared in the study. (**A**). Classical Sforzesco very rigid brace (VRB group). (**B**–**D**). Sforzesco very-rigid brace with the Free Pelvis innovation (FBP group). Posterior full brace view (**B**) and focus on the Free Pelvis innovation on the sagittal (**C**) and frontal (**D**) plane.

The concept of very rigid bracing was recently introduced [7] and showed promising results in high-degree surgical AIS curves [8,9]. Unfortunately, it is a common experience that the higher the brace rigidity, the more severe the skin problems, mainly where bones are superficial, such as at pelvis protuberances (e.g., iliac spines and iliac crests). We recently introduced the “Free Pelvis” (FP) innovation into very rigid braces (VRBs) [10], using semi-rigid material (ethylene vinyl acetate) on the pelvis connected to the main very rigid body of the brace (high-density polyethylene) on the trunk (Figure 1). Patients immediately reported increased comfort with the material, less tension due to spontaneous adaptation of the pelvis according to the new spinal positioning, and a slightly increased pelvic mobility while walking. We noticed FP’s greater adaptability because it allows disentangling the pelvis diameter from the trunk. This is particularly critical for braces having a push-up action because the immediate body adaptations with trunk diameter changes require a progressive tightening of the brace [7]. The pelvis diameter does not change and impairs the continuing action on the trunk. The advantages in comfort and adaptability may make FP an interesting innovation generalizable to other braces. 

We noticed important changes in the brace characteristics and corrective biomechanics during the first months of implementation. Even though we did not perceive different results with or without FP, before further generalization and increased use, we wanted to ensure that the advantages for the patients did not come at the cost of reducing the corrective efficacy. With this aim, we planned a retrospective study to compare the short-term radiographic results (immediate in-brace and first out-of-brace follow-up) [11]) in two matched VRB-treated AIS cohorts with or without FP.

## 2. Materials and Methods

### 2.1. Design 

This was a matched retrospective cohort study. The data were prospectively collected since the development of the Sforzesco brace in a tertiary referral institute specialized in conservative scoliosis treatment. We collected all the available data up to 29 December 2020. The Local Ethics Committee approved the study. We published the protocol on clinicaltrials.gov, 27 April 2021 (National Clinical Trial Identifier Number: NCT04904627). The parents of all patients provided informed consent to the collection of anonymized clinical data for retrospective analysis. The study did not receive any external funding. Reporting of the study follows the STROBE checklist indications [12].

### 2.2. Participants

We included consecutive adolescents according to the following inclusion criteria: diagnosis of AIS, aged 10 to 16, Sforzesco very rigid brace prescribed 23 h/day at the first consultation, frontal radiographs available at three time points considered (baseline, in-brace, and out-of-brace at first 6 months medical follow-up), primary curve above 35°, angle of trunk rotation (ATR) above 7° Bunnell, and Risser score (bone formation) incomplete (up to 3). Exclusion criteria were any other previous or current pathology of the spine or neuromuscular and musculoskeletal systems, and any disease possibly associated with scoliosis.

### 2.3. Treatments

We compared the short-term results of patients treated with the same Sforzesco very rigid brace: one group with the Free Pelvis innovation (cases: FPB group) and controls without (VRB group: standard of care). The choice to use the FP innovation was initially performed in autonomy by the orthotist based on the possibility/willingness to test the FP innovation. After a few months, the FPB became the new standard of care, due to the increased comfort of the patients.

The Sforzesco brace [7] (Figure 1A) was the first brace developed using the high rigidity concept recently introduced in the international classification of braces [5]. This feature was obtained using two 4 mm thermoplastic copolyester or 5 mm high-density polyethylene valves covering the two sides of the trunk and connected with aluminum hinges. An aluminum posterior bar was initially used, but it is now not mandatory because it has no real mechanical action. The correction is achieved due to the newly discovered corrective tool of drivers, acting on the slopes, pushing up the spine, and correcting the frontal and horizontal planes while keeping the sagittal physiological curves [7]. The attention to the sagittal plane is one of the major features of the Sforzesco brace. The very high rigidity achieves the same corrective results as the Risser cast [13] and superior results compare with the Lyon brace [14]; end-of-growth results have also been documented according to classical indications [6] and in extreme, surgical cases refusing spinal fusion [8]. 

The FP innovation [10] (Figure 1B–D) substitutes the use of high-density polyethylene on the pelvis with semi-rigid material (ethylene vinyl acetate) while keeping all the other features of the VRB. According to the current classification of braces [5], the two braces compared in this study are similar: overall action: push-up; anatomical type: TLSO (thoraco-lumbo-sacral orthosis); material: very-rigid; main plane: three-dimensional; and construction: bivalve with anterior closure. 

More details on the clinical approach are reported elsewhere [6]. In this study, all patients had to wear the brace 23 h per day. From 2010, all patients were also prescribed the use of a Thermobrace temperature sensor to verify adherence to treatment [15]. They were also prescribed physiotherapeutic scoliosis specific exercises (PSSEs) according to the Scientific Exercises Approach to Scoliosis (SEAS) school [16]: they were performed without the brace, apart from individual needs as per the physiotherapist’s decision. A cognitive-behavioral approach was followed by all the team to support patients and families. All clinicians (physicians, orthotists, and physiotherapists) respected the SOSORT criteria for bracing management [1,17].

### 2.4. Outcome

The primary outcomes included the Cobb degree variations of the major curves measured on coronal radiographs. We had three observation times: at baseline (T0), in-brace (T1), and out-of-brace (T2). The in-brace radiograph was performed after one month of brace wearing (T1). The out-of-brace radiograph was performed immediately after brace removal at the first consultation six months after prescription (T2). We previously verified the reliability of the radiographic measurements by clinicians in our institute [6,18]. The secondary outcomes included clinical assessment of the angle of trunk rotation (ATR), measured using a Bunnell scoliometer [19] and aesthetics using the Trunk Aesthetic Clinical Evaluation (TRACE) score [20]. The treating physicians collected all measurements at baseline and at the first visit after 6 months of bracing.

### 2.5. Matching

**Figure 2:**
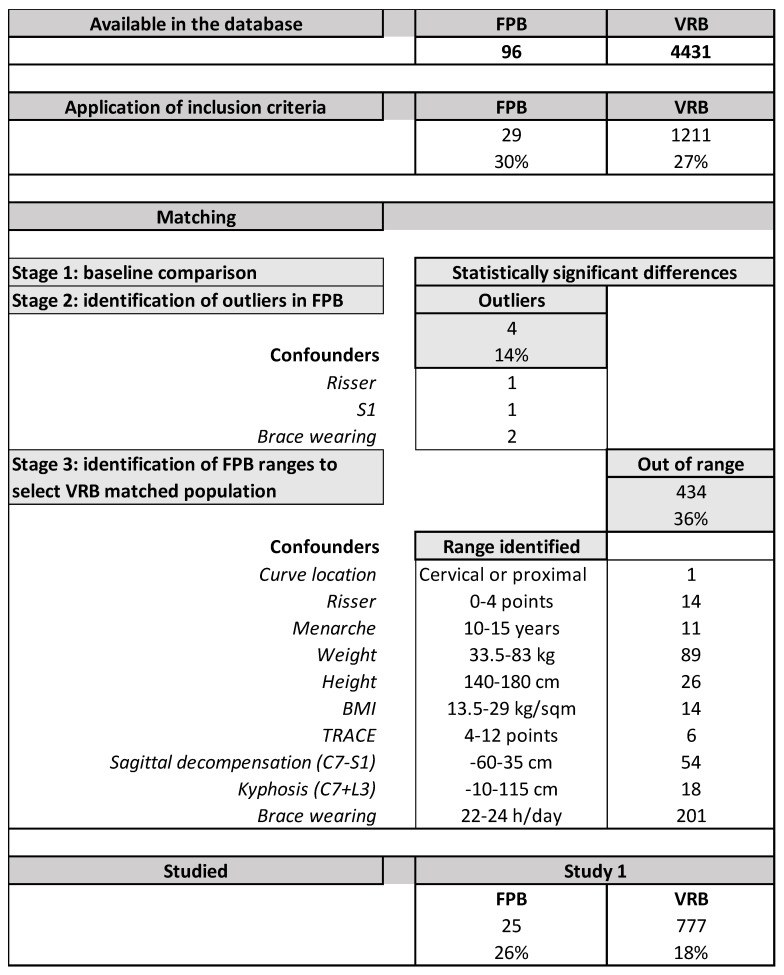
Flow chart of population selection from the clinical data charts.

We hypothesized that there would be no differences in the corrective effect of the classical (VRB group) and the modified Sforzesco brace using the FP innovation (FPB group). We considered the following possible confounders: age at menarche, brace dosage referred or objectively measured [15], curve localization and clinical measures of the ATR [19] and aesthetics (TRACE) [20], sagittal profile measured with the sagittal index [21], the sagittal decompensation (C7 − S1 plumblines), weight, height, and BMI. We performed a matching procedure to ensure equal distribution of confounders in subjects treated with VRB and FPB. The matching could reflect the wide variability in subjects with AIS due to the large population treated with VRB since 2005. We matched the small FPB cohort to the large VRB cohort using the abovementioned confounders. We followed a multiple-stages process (Figure 2): (1) we compared the baseline data to check if there were differences in confounders between the groups; (2) we excluded all the outliers in FPB identified by a value of a confounder above two standard deviations from the average; (3) we used the range of values of patients remaining in the FPB cohort to select the VRB cohort; (4) we compared the resulting samples to check residual baseline differences between the two populations. In case of clinically important baseline differences, we planned subgroup analysis to check if the potential confounder changed the final results.

### 2.6. Statistical Analysis

After checking the normal distribution of the data, we described participants with proportion, mean, and standard deviation. We used paired and unpaired t-tests to check variations between and within brace groups. We tested the brace type as a predictor of results with prediction modeling. We used linear regression to test predictors of the variation in major curve Cobb degrees from baseline to in-brace and short-term (first out-of-brace) radiographs, and logistic regression to test predictors of improvement from baseline to short-term of the major curve exceeding the measurement error of 5 Cobb degrees. 

A univariate linear regression with a change in Cobb angle of the major curve from baseline to short-term guided the choice of the covariates to be included in the final model. We tested the following covariates: brace group, sex, menarche as a binary variable (yes/no), age at start and at menarche as a continuous variable, prior treatment (binary variable, yes/no), ATR, TRACE, sagittal index, BMI, Risser at baseline, and declared and measured dosage of brace wear as a continuous variable. We checked the odds of worsening or improving with logistic regression, with brace type as the explanatory variable, and the same covariates were tested in the linear regression model. The univariate model guided the choice of the covariate to be included in the multivariate model. We ran a sensitivity analysis to check the results after excluding patients differing in baseline characteristics.

The alpha level of significance was set at 0.05. For the analysis, we used STATA 15 software (Copyright 1985–2017 StataCorp LLC, College Station, TX 77845, USA).

## 3. Results

At the time of our study, we found a total of 96 and 4431 patients in the FBP and VRB groups in the database, respectively. Twenty-seven percent met the inclusion criteria. At the start of the matching process, we excluded four outliers in the FPB group because they had data above two standard deviations from the average of the group for Risser sign (1), anterior decompensation (1), or brace wearing time (2). We then looked at the range of each single confounder of the remaining FPB group participants to select the final VRB group, excluding 434 patients (36%), mostly due to brace wearing time (201), anthropometric measures (129), and sagittal decompensation (54) (Figure 2).

We finally included 777 VRB and 25 FPB patients, age 13 ± 1 and 13 ± 1 years, 47° ± 7° and 48° ± 10° Cobb, 11% and 16% men, respectively (Figure 2). At baseline, we did not find a statistically significant difference between the groups for most of the confounders, with three exceptions (Table 1). There was a difference in the number of patients braced before the first consultation (+26% in VRB) that could be clinically meaningful; consequently, we checked the results post hoc, excluding the patients previously braced. We kept the whole sample because we did not find differences in the results, as confirmed by the sensitivity analysis. We also found a statically significant difference in brace wearing time, with FPB patients reporting +12′ per day, and 1% more recorded compliance than VRB. Because these differences were not clinically significant, we did not perform any further analysis.

**Figure 3:**
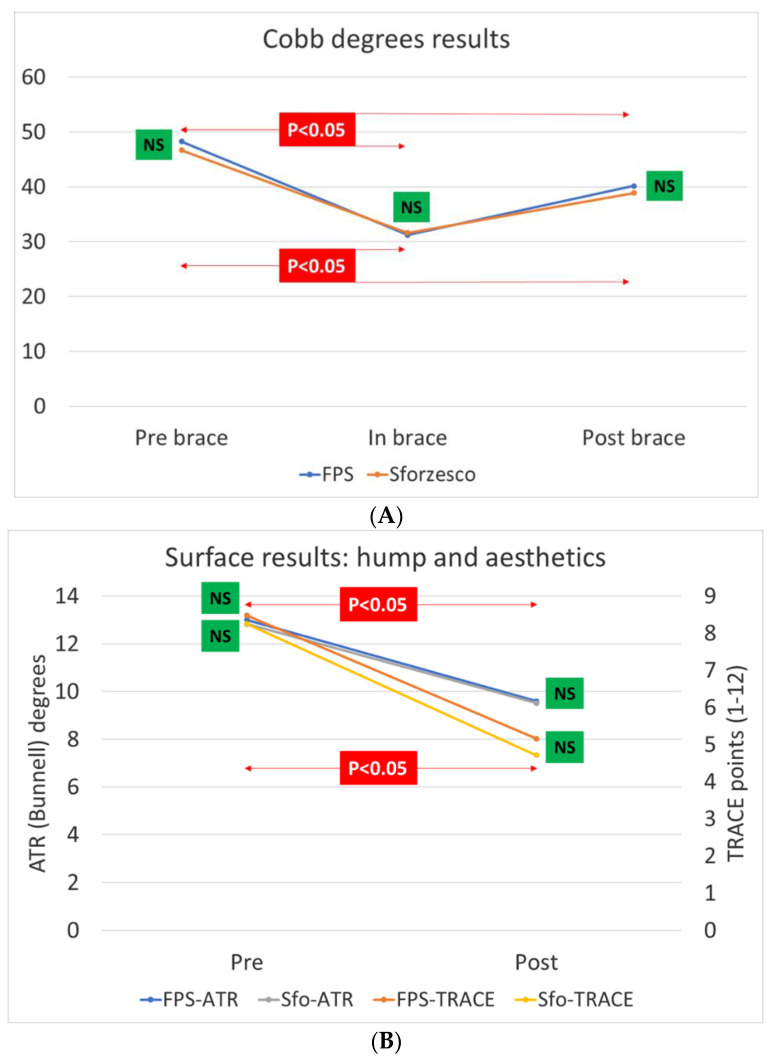
Main results pf this study. We did not find differences at any time stage for the primary Cobb degree outcome (**A**) or at the start and the end for the secondary angle of trunk rotation (ATR degrees) and aesthetics (TRACE points) outcomes (**B**).

The treatment lasted 5 ± 1 months, with no difference between the groups. For the primary outcomes (Figure 3), we observed a statistically significant Cobb degree improvements for VRB and FPB of 15.2 ± 7.7 and 17.4 ± 6.5 at in-brace time point (T1) and of 7.8 ± 0.2 and 8.1 ± 1.3 at T2. The secondary outcomes, ATR and aesthetics, statistically improved in both groups with no intergroup differences (Figure 3).

Brace type, prior treatment, and TRACE were significant in the univariate model, and we tested them in the multivariate model. The type of brace did not influence Cobb degree results either at T1 (coefficient 2.2; 95% CI −0.64/5.1; R^2^ = 0.002) or at T2 (coefficient −0.30; 95% CI −2.4/1.8; R^2^ = 0.0001). The percentage of patients who improved or progressed for all outcomes was not statistically different between the groups (Table 2). Brace type did not affect odds of improvement (OR 0.60; 95% CI 0.3/1.4; adj R^2^ = 0.002).

## 4. Discussion

We recently introduced the FP innovation in very rigid bracing to increase patient comfort and brace adaptability. Before extending the use of this innovation, we felt the need to check its safety and test its effectiveness compared with the standard of care. This study did not find immediate or short-term radiographical and clinical differences between the two matched retrospective cohorts of adolescents with idiopathic scoliosis treated with VRB with or without FP. The present results encourage the continued use of the FP innovation in clinics and studying and monitoring it in broader cohorts until the end of growth. Further implementation will also allow the study of other variables such as compliance, skin problems, number of braces needed, sagittal balance, and back pain. Because this innovation is also suitable for other brace designs, other experts may start introducing and studying FP in different types of braces. 

Brace biomechanical action is yet not completely understood, thus impairing the current understanding of bracing [22]. The recently published braces classification grouped them according to expert knowledge [5]. The everyday clinical reality is that each brace has a different action [23] and even if the clinicians may use the same name for a brace. Moreover, the developers of new instruments, such as the recent very rigid ones, Sforzesco [7] and ART braces [8], reported that their biomechanical thinking changed when changing orthoses. Consequently, there is the need to carefully clinically test any innovation such as the FP potentially impacting the biomechanical action in vivo before its routine implementation.

Another issue is the length of treatment, usually lasting from a minimum of 2.5–3 years up to more than a decade in infantile cases [22]. In this context, it is problematic to properly introduce innovations and test their safety. It is clinically, ethically, and scientifically unacceptable to implement new braces or innovations in everyday clinical practice without proper investigation. Safety comes before efficacy and should be studied as soon as possible to decide to continue implementation in clinical practice. The current SOSORT-SRS Guidelines for brace research [11] define specific steps to face the issue of treatment length. Immediate in-brace results are the first answer and have shown their correlation with final results [22]; even more robust is the correlation with the short-term results, usually collected at 6–12 months [24]. Our study followed these suggestions to provide safety information as soon as possible.

The recent classification [5] recognizes that braces are artisan work, and they may be appropriately constructed or not: the name of the brace does not necessarily correspond to the efficacy of the brace [5]. The current guidelines [1,17] stress the importance of the competence of the prescriber (physician) and builder (orthotist). It is very difficult to study competence, even if it is reproducible by respecting specific criteria. This study included only highly expert orthotists and physicians according to the SOSORT criteria [1,17].

These results are generalizable to braces using the push-up concept [5] and an overall three-dimensional action such as those proposed in this paper. One potential concern for braces following other principles is that the Free Pelvis may result in the loss of some brace patient coronal balance control: this did not happen in our experience but may happen in different designs searching for different biomechanical actions. Nevertheless, the greater comfort given to the patient remains a strong push to generalize this innovation.

The main limitation of the study is the small population in which we tested the FP innovation. It was urgent to verify the safety of the introduced innovation. Other limitations were matching with narrow criteria and comparing them to the largest available population treated with standard VRB and compensating for the potential limitation related to the well-known wide variance in AIS patients. Another limitation is the retrospective design. Due to the study design, we also did not perform a sample-size calculation and looked for the widest population possible. Still, this design is the best possible at this early stage of research in terms of cost-effectiveness and fully justified by the need to quickly verify the safety of this innovation. Another issue is the matching confounders: we tried to be as comprehensive as possible, including most of the confounders known in the literature. The short-term results may change over a longer observation time, even if the current literature supports their use [24,25]. The study’s strength is the broad population considered, particularly in the matched sample, which could minimize the risk of poor matching: at baseline, the differences were minimal and not clinically significant. Of interest for future studies is our result of a statistically (albeit not clinically) significant difference in favor of FPB for compliance and brace wearing that may be explained by better comfort.

## 5. Conclusions

We recently introduced the Free Pelvis in very rigid braces for patient comfort and brace adaptability. We found that the FP innovation does not change the in-brace and short-term results of the Sforzesco VRB. Our findings are relevant because short-term results have a prognostic value for the end-of-growth outcomes. The present results also show the safety of the FP innovation and encourage continuing the clinical application, and to study it in broader cohorts. The FP may also be implemented in other brace designs.

## Figures and Tables

**Table 1:** Baseline data with all possible confounders. Management of the statistically significant differences is explained in the text.

		FPB		VRB		
		Average	SD	Average	SD	*p*
Number		25		777		
Males		16%		11%		NS
Age	years	13.3	1.5	13.1	1.5	NS
Risser	score	1.4	1.4	1.7	1.4	NS
Age at menarche	years	11.6	1.3	11.8	1.1	NS
Menarche		64%		66%		NS
Weight	kg	49.4	11.3	49.9	8.8	NS
Height	cm	159.8	8.7	158.7	7.3	NS
BMI	kg/sqm	19.3	3.6	19.8	2.9	NS
Previous brace		20%		46%		0.009
Main curve	Cobb degrees	48.3	10.0	46.7	7.4	NS
Thoracic proximal		4%		1%		NS
Thoracic		64%		71%		
Thoracolumbar		20%		14%		
Lumbar		8%		13%		
Main prominence	ATR degrees	13.0	3.9	12.8	3.4	NS
Aesthetics	TRACE index	8.5	1.9	8.3	1.9	NS
Plumbline C7 + L3	mm	46.4	30.3	46.5	25.6	NS
Plumbline C7 − S1	mm	0.2	20.6	−0.9	17.7	NS
Declared brace use		23.1	0.5	22.9	0.4	0.027
Recorded brace use		0.9	0.0	0.9	0.1	0.000

**Table 2:** Main clinical results in the two studied cohorts.

		FPB (N = 25)			VRB (N = 777)			*p*
		Improved	Unchanged	Progressed	Improved	Unchanged	Progressed	
Main curve	Cobb degrees	68%	32%	0%	77%	23%	1%	NS
Main prominence	ATR degrees	60%	40%	0%	60%	40%	2%	NS
Aesthetics	TRACE index	68%	32%	0%	70%	30%	0%	NS

## Data Availability

The data presented in this study are openly available from Zenodo at https://doi.org/10.5281/zenodo.6469615 (accessed on April 2022).
